# Visfatin as a predictor for growth of fetus and infant

**DOI:** 10.4274/tjod.48091

**Published:** 2018-06-21

**Authors:** Ashraf Saber Mashhad Taraqi, Najmeh Tehranian, Marzieh Faghani Aghoozi, Somayeh Yousefi, Anoshirvan Kazemnejad, Shiva Pourali Roudbaneh, Matin Sadat Esmaeilzadeh

**Affiliations:** 1 North Khorasan University of Medical Sciences Bojnurd Faculty of Nursing and Midwifery, Department of Midwifery, Bojnurd, Iran; 2 Tarbiat Modares University Faculty of Medical Sciences, Department of Midwifery and Reproductive Health, Tehran, Iran; 3 Shahroud University of Medical Sciences Faculty of Nursing and Midwifery, Department of Midwifery, Shahroud, Iran; 4 Birjand University of Medical Sciences Faculty of Nursing and Midwifery, Department of Midwifery, Birjand, Iran; 5 Tarbiat Modares University Faculty of Medical Sciences, Department of Biostatistics, Tehran, Iran; 6 Guilan University of Medical Sciences Faculty of Nursing Midwifery and Paramedicine, Department of Midwifery, Rasht, Iran

**Keywords:** Visfatin, spredictor, grow, fetus, infant

## Abstract

**Objective:**

Visfatin is an adipocytokine that functions as an enzyme and a growth factor to investigate the relationship between serum visfatin and the fetus’s anthropometric markers up to a year after birth.

**Materials and Methods:**

Forty-one eligible pregnant women in their first trimester were divided and matched in terms of body mass index (BMI) before pregnancy into normal and higher than normal BMI groups, A and B. Serum visfatin levels were measured during 6-12 and 15-20 weeks of gestation using ELISA.

**Results:**

The infants were followed up for a mean duration of 10.19±2.83 months. In group A, there was a strong positive relationship between birth head circumference and the first (p_1_=0.054, r_1_=0.580) and second trimester visfatin levels (p_2_=0.051, r_2_=0.530). In group B, second trimester visfatin levels correlated negatively with birth length (p=0.015, r=-0.523) and infant’s head circumference (p_2_=0.050, r_2_=-0.392). In a separate study on group B, visfatin levels in the first and second trimesters showed a significant negative correlation with infant’s weight. A significant correlation was observed between the first and second trimesters visfatin level with infant’s height in both groups, such that this relationship was positive in group A and negative in group B. Linear regression analysis revealed that first and second trimester visfatin levels were significant independent predictors of infant’s weight in group B and infant’s height in both groups. Second trimester visfatin level was a significant predictor of birth height in group B.

**Conclusion:**

Maternal serum visfatin level shows a relationship with fetal and infant anthropometric indicators, with different effects in the two groups, suggesting visfatin dysfunction in the overweight group before pregnancy.

**PRECIS:** We measured the serum visfatin level in pregnancy, and assess its relationship with the children’s anthropometric markers, to determine if it can be used as biomarker.

## Introduction

By releasing adipokines, adipose tissue has a major role in fertility, and physical and sexual maturation^(1)^. Visfatin, a new 52KDa adipokine^(2)^, was renamed to nicotinamide phosphoribosyl transferase (Nampt) in 2002 after it was shown that it encodes an enzyme called Nampt, which is involved in the conversion of nicotinamide into nicotinamide adenine dinucleotide (NAD)^(3)^. Intercellular Nampt is substantially released by embryonic, amniotic, and placental membrane and adipose tissue. In a term fetus, amnion and decida have higher levels of mRNA of the visfatin gene^(4)^. Yet, visfatin’s regulatory and release mechanisms in the fetus and neonate are still unclear^(5)^. It is probably regulated by glucose and insulin^(6)^, and increases with gradual degradation of B-cell and progress of insulin-resistance and maternal weight^(7)^. Many studies have shown that increased visfatin levels in maternal plasma are associated with small-for-gestantional-age births^(8)^ and intrauterine growth restriction^(9)^. Serum visfatin levels fluctuate as pregnancy advances^(10)^. Strong relationships have recently been reported between serum visfatin levels in the first trimester of pregnancy and insulin secretion in the fetus and final birth weight, which shows the role of visfatin secretion in early pregnancy in the later metabolic modeling of the fetus^(11)^. These results suggest that visfatin may play an important role in maternal-fetal metabolic interaction. However, due to the lack of sufficient information about the physiologic role of visfatin in adults, its source and regulation mechanism in fetal and neonatal stages cannot be argued absolutely. To investigate the role of visfatin in the growth of the fetus and up to a year after birth, we measured serum visfatin levels during 6-12 and 15-20 weeks of gestation in an Iranian population.

## Materials and Methods

**Figure 1:**
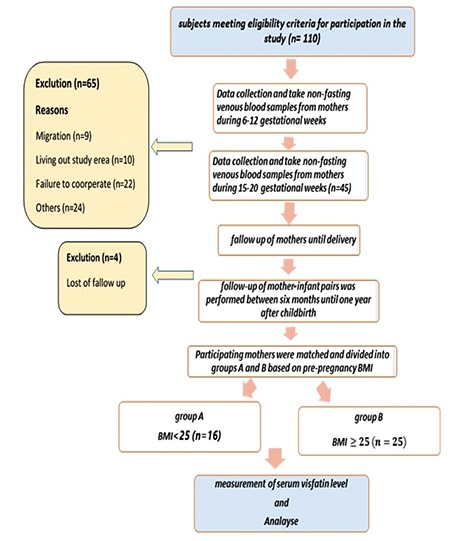
Flow chart of the cohort *BMI: Body mass index*

The present cohort study was conducted between 2013 and 2015 after obtaining approval of the Research Council of Tarbiat Modares University, Tehran, Iran, and permission from the Medical Ethics Committee of the Faculty of Medical Sciences, and presenting a letter of introduction from the university to selected and densely populated medical centers covered by Shahid Beheshti University of Medical Sciences in the north, east, and Shemiranat districts of Tehran, Iran. This study was approved by the Ethics Committee of Tarbiat Modares University (registration number: IR.TMU.REC.1394.120). The study is registered in the Iranian Research Institute for Information Sciences and Technology. A flow chart of the study is presented in Figure 1. First, pregnant women were briefed on the study objectives and the confidentiality of maternal and neonatal information, and they then signed informed consents. Then, 41 eligible pregnant women in their first trimester were selected through convenience sampling. The study inclusion criteria were age 18-40 years, singleton pregnancy, no systemic diseases such as lupus and diabetes mellitus, and Iranian nationality. The study exclusion criteria were pregnancy complications (e.g., diabetes, preeclampsia), psychological problems, tobacco or alcohol use in the first trimester of pregnancy, medications other than pregnancy supplements, abnormal stresses such as family bereavement and accidents, migration, living outside of the study area, failure to cooperate, follow-up exitus, and becoming pregnant again within a year. Demographic details, pregnancy history and maternal medical history were found through direct interviews with mothers in their first pregnancy visit, based on the ministry of health’s routine prenatal care questionnaire. Gestational age was calculated based on the first day of the last menstruation (LMP), or the first trimester ultrasound (if uncertain about LMP). Weight, blood pressure, and heart rate of the fetus were measured by the same person using a digital scale, digital barometer, and fetal heart detector (sonicaid). At the end of their visit, mothers received training on proper pregnancy nutrition. The researcher took non-fasting venous blood samples from mothers during 6-12 and 15-20 gestational weeks between 09:00 and 11:00 am. The samples were added to ethylenediaminetetraacetic acid-containing tubes and sent to endocrinology and metabolism laboratories of Shahid Beheshti University of Medical Sciences, Tehran within 24 hours and kept at 2-8 °C. For plasma separation, samples were centrifuged at 3000 rpm and 4 °C for 10 minutes. The resulting plasma was frozen at a temperature of ≤-20 °C until required for analysis. Anthropometric indicators (birth height, weight, and head circumference) were measured. The next follow-up of infants was performed between six months and one year after childbirth (both cesarean and vaginal) through telephone contact with the mothers, during which they were asked to attend their local health centers for the assessment of anthropometric indicators (growth monitoring curve, height, weight, and head circumference) and also neonatal diseases. Children were assessed based on the ministry of health’s questionnaire for children under 8 years of age. Afterwards, participating mothers were matched and divided into groups A and B based on pre-pregnancy body mass index (BMI) as follows: normal (18.5-25 kg/m^2^) (n=16) and overweight and obese (≥25 kg/m^2^) (n=25). Pre-pregnancy BMI based on pre-pregnancy weight was calculated using the following equation as defined by the World Health Organization: weight/height^2^. BMI between 18.5 and 25 kg/m^2^ is considered normal, and ≥25 kg/m^2^ is abnormal.

### Statistical Analysis

The homogeneity of the two groups was confirmed through statistical tests in terms of underlying and demographic variables such as age, education, employment, household size, and neonatal sex, fertility status, income, childbirth method, and family planning, the time presenting for postpartum childcare, feeding method (formula, breast milk, or both), hypothyroidism, neonatal anemia, and referral to physician. Serum visfatin levels were measured using enzyme-linked immunosorbent assay (ELISA) with human visfatin (ZellBio GmbH, Germany; Ulm kit, Cat No: ZB-3408-H9648). Finally, visfatin levels were compared using data from maternal and neonatal anthropometric measurements. Normal distribution of variables in each group was assessed using the Kolmogorov-Smirnov test. The independent and paired t-test were used for normally distributed quantitative variables, the chi-square test was used for qualitative variables, and the Mann-Whitney U and Wilcoxon Signed-Rank tests were used for non-normally distributed variables. The relationships among the present study variables in each group were separately assessed using independent t, Pearson’s, linear regression, and Spearman’s ordinal correlation tests. Finally, lambda stat was used to compare correlation coefficients of variables in the two groups at p<0.05.

## Results

Demographic and underlying variables from the two groups are presented in Table 1.

### Variations in visfatin level during pregnancy

Mean non-fasting serum visfatin level of participating mothers (n=41) was 59.4±68.01 ng/mL (range, 4.9-234 ng/mL) in the first trimester, and 76.98±75.55 ng/mL (range, 4.6-248 ng/mL) in the second trimester, and variations in visfatin level were reported as 17.58±26.46 ng/mL.

### The relationship between visfatin level and neonatal anthropometric indicators at birth

In group A, a strong positive relationship was observed between birth head circumference and the first (p_1_=0.05, r_1_=0.580) and second trimester visfatin levels (p_2_=0.051, r_2_=0.530). Spearman Rank correlation test showed a negative significant relationship between second trimester visfatin level and birth height (p=0.015, r=-0.523) in group B (Table 2). Linear regression analysis revealed that the second trimester visfatin level was a significant predictor of birth height in group B (Table 3).

### The relationship between visfatin level and infant’s anthropometric indicators

The mean duration of neonatal follow-up was 10.19±2.83 months. Significant correlations were observed in both groups between first trimester visfatin (p_a_=0.002, r_a_=0.713, p_b_=0.005, r_b_=-0.540, in A and B group, respectively) and second trimester visfatin (p_a_=0.009, r_a_=0.628 and p_b_=0.008, r_b_=-0.518, in groups A and B, respectively) with infant’s height. This correlation was positive in group A and negative in group B. According to the Lambda test, this correlation was significantly greater in group A than in group B (p<0.05). A negative and significant correlation was separately found between infant’s weight and the first (p_1_=0.024, r_1_=-0.450) and second trimester visfatin levels (p_2_=0.005, r_2_=-0.540) in group B. Generally, the relationship between visfatin and the infants’s growth indicators was positive in group A and negative in group B. A strong negative correlation was separately found between the second trimester visfatin level and infant’s head circumference (p_2_=0.05, r_2_=-0.392) in group B (Table 2). Linear regression analysis revealed that first and second trimester visfatin levels were significant predictors of infant’s weight in group B and infant’s height in both groups (Table 3).

## Discussion

No study has yet been conducted to assess the relationship between maternal serum visfatin and neonatal anthropometric indicators by categorizing mothers based on BMI. The visfatin regulation mechanism in human fetal blood circulation is unknown^(12)^. However, recent studies have proposed the presence of a relationship between maternal serum visfatin level and fetal growth. A strong relationship was found between maternal serum visfatin and neonatal anthropometric indicators in groups A and B, with a possible mechanism of maternal visfatin entering the fetal blood circulation through the placenta as a result of endocrine changes. In a study by Cekmez et al.^(13)^, anthropometric indicators, including weight and height, were measured and plasma lipids, insulin, and adiponectin and visfatin concentrations in the umbilical cord blood of 50 large-for-gestational age (LGA) and 50 appropriate-for-gestational age (AGA) infants born following complication-free pregnancies were assessed. The mean visfatin and adiponectin levels were significantly higher in the macrosomia group compared with the AGA group. Moreover, umbilical cord serum visfatin concentrations were found to have a positive relationship with insulin levels^(13)^. Mazaki-Tovi analyzed the relationship of increased maternal serum visfatin with gestational diabetes mellitus infection and the birth of LGA infants. An increase in maternal blood glucose leads to a rise in fetal blood glucose, which in turn stimulates fetal pancreatic islet cells, resulting in hyperinsulinemia. In LGA infants, hyperinsulinemia in fetal period leads to fetal macrosomia^(14)^. One of the reasons for a visfatin effect on fetal development is its effect on sirtuins (SIRT). SIRTs are a class of proteins that have a role in cellular processes associated with body metabolism such as cell differentiation, aging, transcription, apoptosis, inflammation, and stress resistance, as well as energy efficiency and alertness during low-calorie situations. Humans have seven  SIRT isoforms. SIRT 1 (SIRT1) affects chromatin modulation, and therefore suppresses transcription and interacts with transcription factors, and is capable of positive or negative regulation of gene expression^(15)^. Through involvement in NAD^+^ synthesis needed for SIRT activity, visfatin has a major role in the regulation of SIRT1-dependent transcription, and this leads to the metabolism of energy and differentiation of stem cells. SIRT Nampt expression increased in both cell models in the course of bone tissue differentiation from multipotent fibroblast and monopotent pre-osteoblast in rats. A rise in Nampt leads to higher concentrations of NAD^+^ and higher activity of SIRT1. In contrast, a reduction or inhibition of Nampt leads to reduced NAD^+^ concentration and SIRT1 activity, resulting in inhibition of osteocyte differentiation. This means that Nampt promotes osteocyte differentiation through a pathway mediated by SIRT1^(16,17)^. High levels of SIRT1 expression in the human brain have been revealed^(18)^. The early expansion of neurons initiates with neurite process elongation pursued by axon differentiation, dendritic arborization, and synapse formation.  SIRTs have an important role in the process of synapse promotion and modulation of their strength, which is important for memory formation. During neuronal development, SIRT1 has a important role in promoting xonal elongation, neurite outgrowth, and dendritic branching via several targets and mechanisms^(19)^. Increasion of nerve growth factor in PC12 cells by Cytoplasmic SIRT1 Induced neuritogenesis^(18)^. The NAD^+^ dependent deacetylase SIRT1 is implicated in energy balance regulation by its effect on pro-opiomelanocortin and agouti-related peptide (AgRP) neurons in the arcuate nucleus of the hypothalamusthis^(20)^. Reduction of energy intake induced SIRT1 in brain^(21)^. Obesity is correlated with low NAD^+^/SIRT pathway expression in adipose tissue of BMI-discordant monozygotic twins^(22)^. The selective knock-out of SIRT1 in hypothalamic AgRP neurons diminished response to hunger-inducing hormone ghrelin, reduced food intake, consequently causing decreased lean mass and body weight^(23)^. Visfatin is expressed in bovine mammary epithelial cells, lactating mammary gland, and milk, which is regulated by the cAMP pathway^(24)^. The persistence of the relationship between postpartum maternal serum visfatin levels and anthropometric indicators can be attributed to excretion of visfatin in mother’s milk, which was confirmed by Bienertová-Vašků et al.^(25)^. They examined milk and venous blood samples of 24 healthy breastfeeding women with complication-free physiologic pregnancies and neonates with appropriate weight for gestational age at birth and 180 days after birth. Visfatin had been copiously excreted in mother’s milk, and serum visfatin levels were 100 times higher in mothers’ milk than in their blood. Significant changes occurred in maternal serum visfatin levels after childbirth. Based on maternal colostrum visfatin levels, the aurors were able to predict neonatal weight loss in the first three days of birth^(25,26)^. Visfatin by effect on the production of SIRT1, consequently, interference in the development of the brain and energy balance regulation may be associated with head circumferences and weights of fetus and child. According to these results, visfatin can have a major role in regulating neonatal obesity after birth. The above studies confirm the present study results. Generally, these results show that visfatin has a major role in maternal-fetal metabolic interaction. Our results further show different metabolic regulations in groups A and B, which may be due to differences in expression of SIRT1, glucose transfer or insulin resistance as a result of resistance to glucose entry to fetal tissues for unknown reasons, which require further studies. It is likely that visfatin can be used in the future as a biomarker predicting fetal and neonatal growth.

### Study Limitations

This study provides useful data of the relationship between serum visfatin and the children’s anthropometric markers up to a year after birth. However, the present study has some limitations. First, we know that it would be better to evaluate levels of cord plasma and breast milk concentrations of visfatin and assess their correlation with the children’s anthropometric in order to support our results. However, we could not do it, which is a limitation of our study. Second, the fallow up of mothers and infants was short-term. We suggest the long-term fallow up of mothers and infants with more abundant sample.

## Conclusion

After a careful review of previous studies on visfatin and pregnancy, we can claim the present study is the first in Iran to have specifically addressed the relationship between maternal visfatin levels and infant’s anthropometric indicators up to a year after birth. Our study can be the basis for further and more precise studies. According to the present study results, mean serum visfatin levels were higher in the second trimester compared with the first. The mean maternal serum visfatin level during pregnancy significantly increased with increasing gestational age in parallel to weight gain and insulin resistance in both groups separately. In the present study, the relationship between prenatal visfatin level and infant’s anthropometric indicators in the two years following childbirth was assessed. Serum visfatin level appears to be related to the fetus and infant’s anthropometric indicators (infant’s weight, height and birth height. First and second trimester visfatin levels were significant independent predictors of infant’s weight in group B and infant’s height in both groups. The second trimester visfatin level was a significant predictor of birth height in group B. Visfatin has extensive effects on pregnancy physiology and pathology, maternal and especially neonatal outcomes, and its effect is different in normal weight and overweight women. Hence, pre-pregnancy BMI appears to be a determining factor in creating the difference and the amount of maternal plasma visfatin during pregnancy, which can predict maternal serum visfatin and neonatal and maternal outcomes. It is recommended that future studies be conducted with larger sample sizes and longer follow-up periods.

## Figures and Tables

**Table 1:**
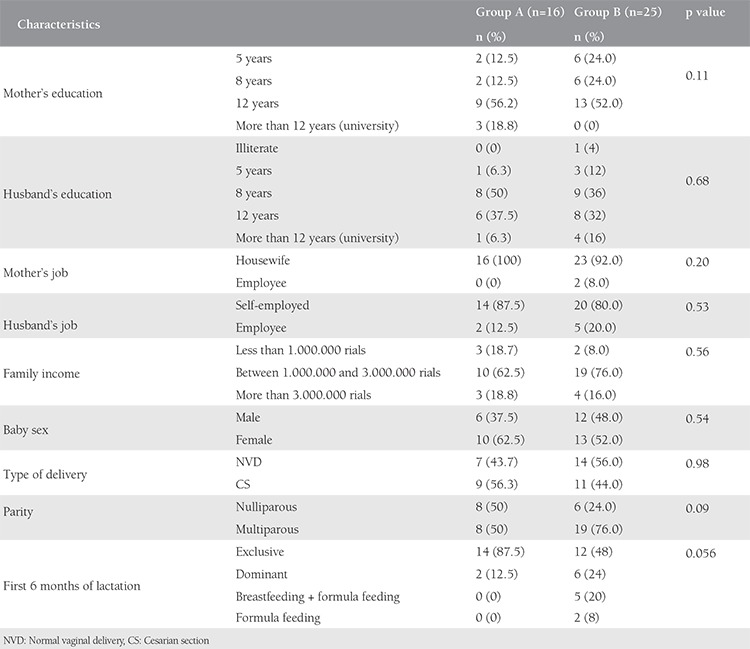
Demographic and clinical characteristics of pregnant women (n=41)

**Table 2:**
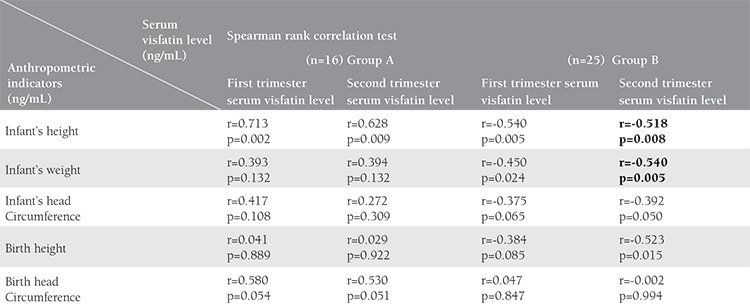
The relationship between visfatin level and anthropometric indicators

**Table 3:**
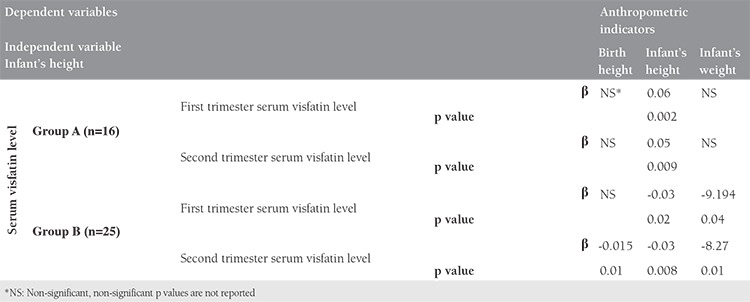
Lenear regression analysis between anthropometric indicators as dependent variable and serum visfatin level
